# Supplementary material for: Butyrate preserves entorhinal-hippocampal spatial coding and blood brain barrier integrity in mice with depleted gut microbiome
Source: bioRxiv. 2025 Jul 31:2025.07.24.666609. Preprint. [Version 1] doi: 10.1101/2025.07.24.666609 (PMC12324207; doi:10.1101/2025.07.24.666609)
Supplement: Supplement 1 [file NIHPP2025.07.24.666609v1-supplement-1.pdf]

bioRxiv preprint doi: https://doi.org/10.1101/2025.07.24.666609; this version posted July 31, 2025. The copyright holder for this preprint (which was not certified by peer review) is the author/funder, who has granted bioRxiv a license to display the preprint in perpetuity. It is made available under aCC-BY 4.0 International license.

Supplementary Materials

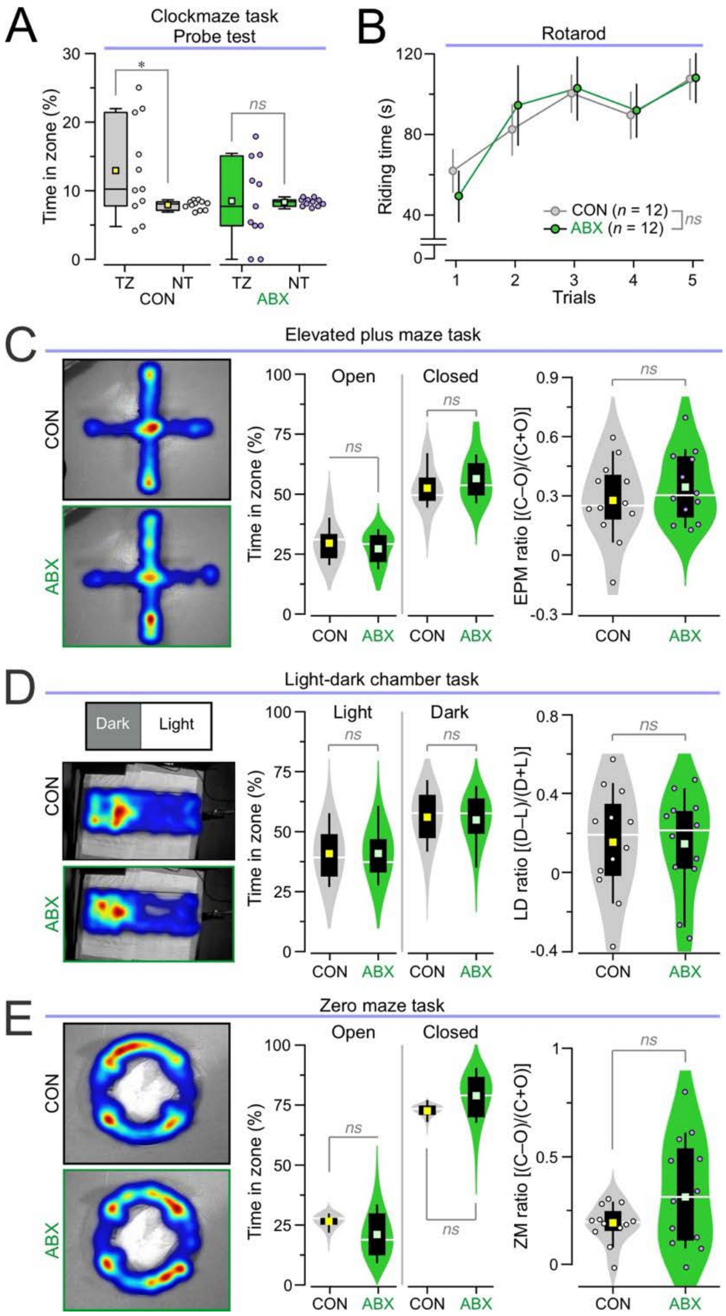

Supplemental Fig. 1. Behavioral tasks in CON and ABX mice. (A) Probe test in the clockmaze task: Box-and-whisker plots for the percent of time in the target zone (TZ) or the non-target zone (NZ). CON mice spend significantly more time in the TZ than the NZ (**P* < 0.031, *t* test, *n* = 11) whereas ABX mice do not show significant difference between zones (*P* = 0.925, *t* test, *n* = 11). (**B**) Rotarod task: dot and line plot showing no differences in riding time across 5 trials in CON and ABX mice (*P* = 0.949, RMANOVA followed by Bonferroni test; *n* = 12 CON, 12 ABX). (**C**) Elevated plus maze (EPM) task: *Left,* representative occupancy heatmaps; *middle,* violin plots with the percent time spent in either the open (*P* = 0.436, *t* test) and closed arms (*P* = 0.305, *t* test); *right,* violin plot with the EPM ratio (*P* = 0.394, *t* test), defined as the time in the closed arms minus the time in the open arms divided by the summed time in both arms, for both groups (*n* = 12 CON, 12 ABX). (**D**) Light-dark (LD) chamber task: *Left,* occupancy heatmaps; *middle,* violin plots with the percent time spent in either the light (*P* = 0.98, *t* test) or dark (*P* = 0.836, *t* test) zones; *right,* violin plot showing the LD ratio (*P* = 0.931, *t* test), defined as the time in the dark zone minus the time in the light zone divided by the time in both zones, for both groups (*n* = 12 CON, 12 ABX). (**E**) Zero maze (ZM) task: *Left,* occupancy heatmaps; *middle,* violin plots with the percent time spent in either the closed (*P* = 0.07, *t* test) or open (*P* = 0.083, *t* test) zones; *right,* violin plot showing the ZM ratio (*P* = 0.071, *t* test), defined as the time in the closed zone minus the time in the open zone divided by the time in both zones, for both groups (*n* = 12 CON, 12 ABX).

bioRxiv preprint doi: https://doi.org/10.1101/2025.07.24.666609; this version posted July 31, 2025. The copyright holder for this preprint (which was not certified by peer review) is the author/funder, who has granted bioRxiv a license to display the preprint in perpetuity. It is made available under aCC-BY 4.0 International license.

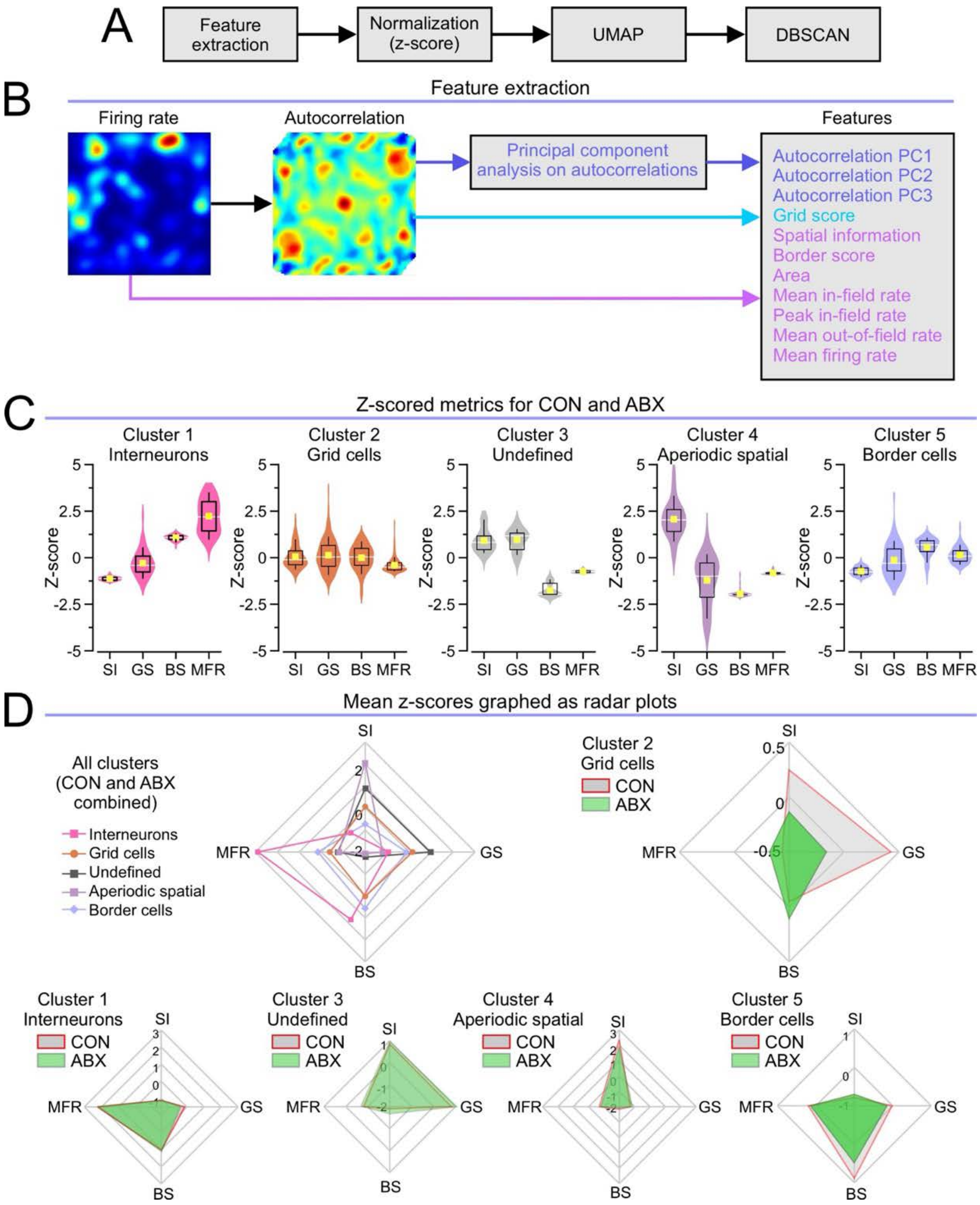

Supplemental Fig. 2. Medial entorhinal cortex neurophysiology analysis pipeline. (A) Diagram outlining major steps in unsupervised learning pipeline. For each isolated neuron, features are extracted and normalized by taking the z-score for each parameter. These normalized features are inputted into a UMAP for dimensionality reduction. The dimensionality reduced data is then clustered using DBSCAN to classify each cell type. (**B**) Diagram explaining feature extraction. Firing rate maps and autocorrelations are generated for all cells. From the rate map, we calculate the following features: SI, border score, area, mean in-field rate, peak in-field rate, mean out-of-field rate, and MFR. From the autocorrelation, we calculate the following features: grid score and the first three components of the principal component analysis. This pipeline is applied separately for two datasets (dataset 1: CON and ABX; dataset 2: BA and ABXBA). All of the metrics above are normalized, and the z-scores are used for the rest of the analysis. (**C**) Violin plots showing z-scored metrics for cluster classification. Distinct clusters emerge in each dataset, characterized by their defining features. They are identified as: interneurons (cluster 1, high MFR, low SI), grid cells (cluster 2, high grid score), undefined (cluster 3, mid-range SI and grid score, low MFR and border score), aperiodic spatial (cluster 4, high SI, low grid score), and border cells (cluster 5, high border score). (**D**) Radar plots showing the mean SI, grid score, border score, and MFR for each cluster. In the top left plot, the means are shown for each cluster with CON and ABX mice combined. The remaining plots show each cluster segregated into CON and ABX mice. Remarkably, cluster 2 for grid cells demonstrates that CON and ABX groups can be easily discriminated.

bioRxiv preprint doi: https://doi.org/10.1101/2025.07.24.666609; this version posted July 31, 2025. The copyright holder for this preprint (which was not certified by peer review) is the author/funder, who has granted bioRxiv a license to display the preprint in perpetuity. It is made available under aCC-BY 4.0 International license.

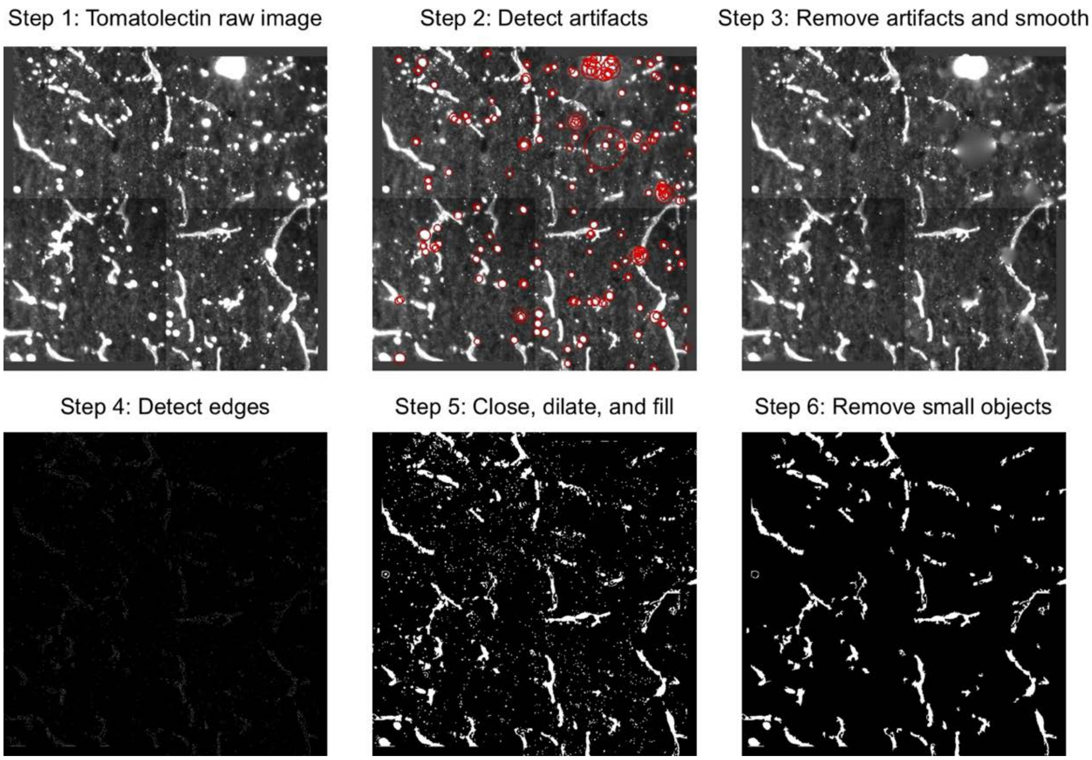

Supplemental Figure 3. Image processing for blood vessel analysis. To determine the percent coverage of occludin and claudin-5 throughout the blood vessels, we create a mask of the tomatolectin stain with a six-step process. In **step 1**, the raw greyscale image of the tomatolectin stain is acquired. In **step 2**, we detect artifacts which likely correspond to aggregated antibody and are clearly distinct from blood vessels by searching for circular objects which are brighter than their surroundings. In **step 3**, these artifacts are removed through interpolation of the surrounding areas, and the image is smoothed with a 2-dimensional Gaussian kernel. In **step 4**, the edges are detected, excluding those corresponding to the previously identified artifacts, converting the greyscale image to a binary black-and-white image. In **step 5**, the blood vessel outlines are defined using a series of morphological dilation and erosion operations (which respectively expand and shrink the detected edges to create continuous vessel boundaries). These outlines are then filled through interpolation. In **step 6**, objects smaller than 1000 pixels are removed to account for image noise, generating the final mask that is used for subsequent analysis.

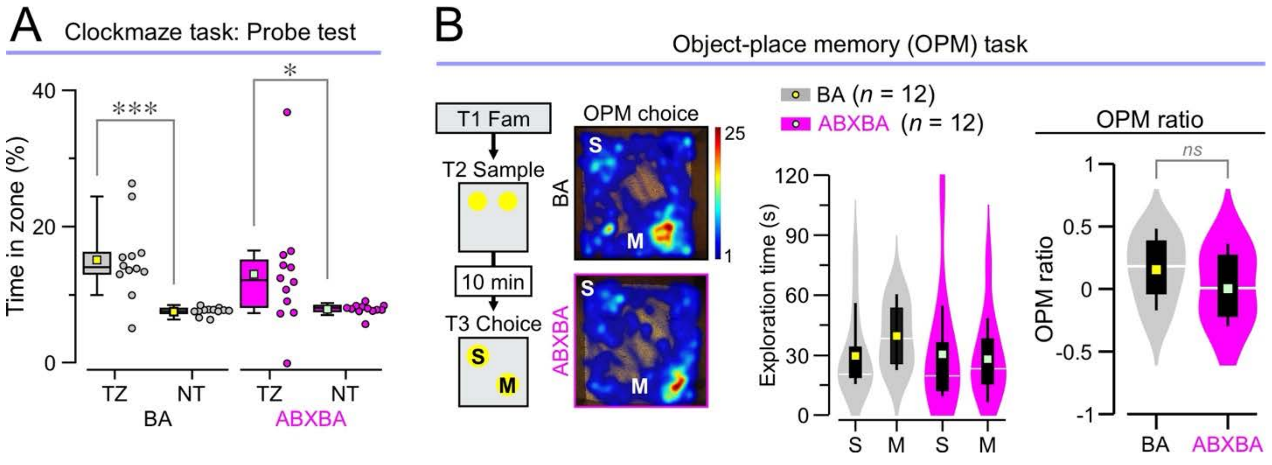

Supplemental Figure 4. Behavioral tasks in BA and ABXBA mice. (A) Probe test in the clockmaze task: Box-and-whisker plots for the percent of time spent in the target zone (TZ) or the non-target zone (NZ). Both groups spend significantly more time in the TZ than the NZ (BA: ***P = 0.00059, Mann-Whitney U test, n = 12; ABXBA: *P < 0.035, Mann-Whitney U test, n = 12). (**B)** *Left,* schematic of the OPM task and representative occupancy heatmaps. *Middle,* violin plots showing the investigation times for the stable and moved objects (BA v. ABXBA, P = 0.404; stable v. moved, P = 0.561, two-way ANOVA followed by Tukey test; n = 12 BA, 12 ABXBA). *Right,* Violin plots of the OPM ratios (P = 0.234, t test; n = 12 BA, 12 ABXBA).

bioRxiv preprint doi: https://doi.org/10.1101/2025.07.24.666609; this version posted July 31, 2025. The copyright holder for this preprint (which was not certified by peer review) is the author/funder, who has granted bioRxiv a license to display the preprint in perpetuity. It is made available under aCC-BY 4.0 International license.

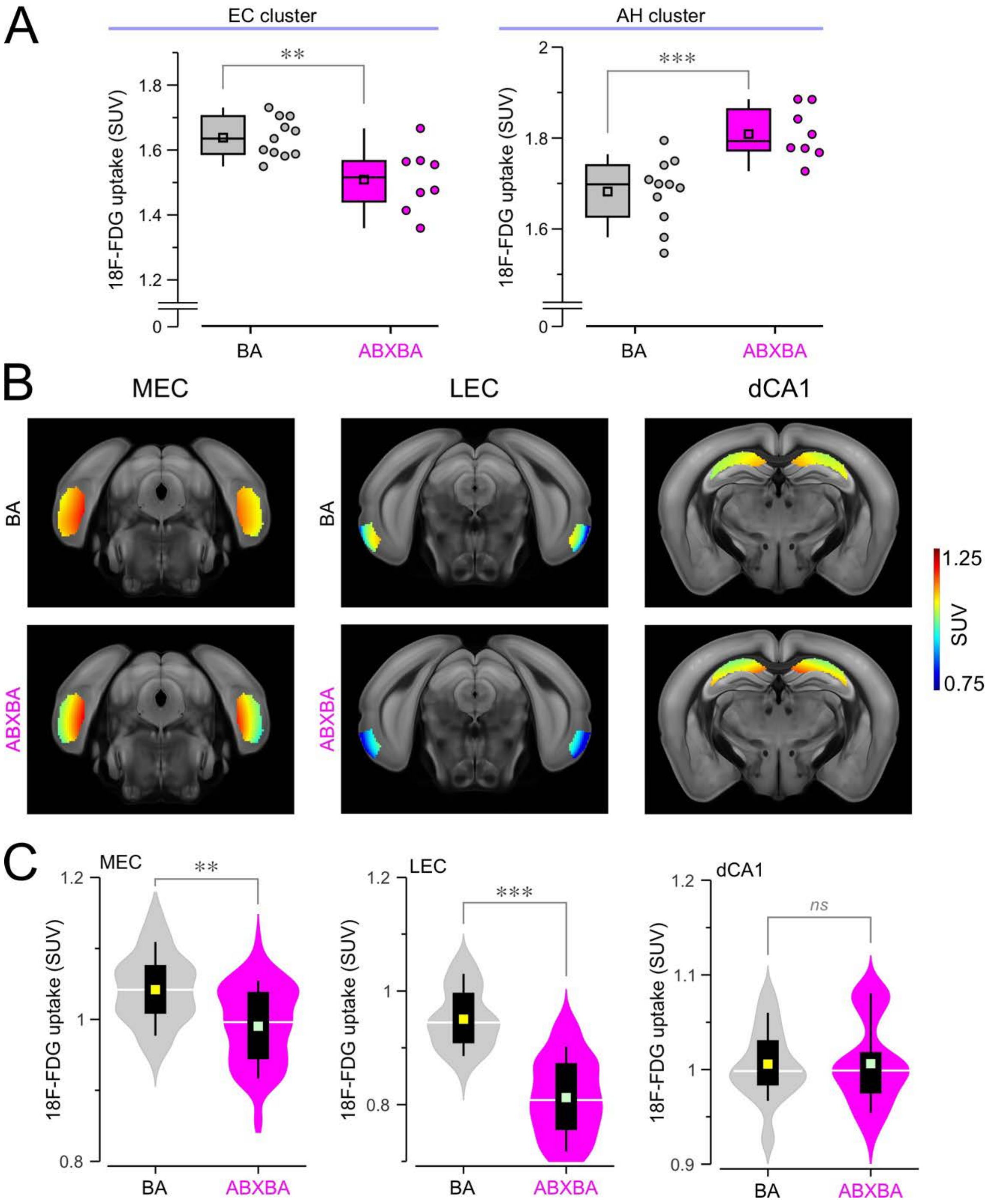

Supplemental Figure 5. Positron emission tomography in butyrate-treated mice. (A) Box-and-whisker plots showing the FDG SUVs for the clusters corresponding to the entorhinal cortex (EC) (*left*) and the anterior hypothalamus (AH) (*right*). Cluster coordinates correspond to those described for CON and ABX mice (Fig. 2, A and B). **(B)** Brain atlas-based analysis with masked slices for the medial entorhinal cortex (MEC), lateral entorhinal cortex (LEC) and dorsal CA1 (dCA1). The heatmaps show the SUVs for one slice averaged across all mice and overlaid onto an MRI template. **(C)** Violin plots showing that ABXBA mice display significantly decreased FDG uptake in the MEC (***P* = 0.001, MMANOVA; *n* = 330 slices from 11 BA, 240 slices from 8 ABXBA), LEC (****P* < 0.001, MMANOVA; *n* = 220 slices from 11 BA, 160 slices from 8 ABXBA), and dCA1 (*P* = 0.944, MMANOVA; *n* = 330 slices from 11 BA, 240 slices from 8 ABXBA).

bioRxiv preprint doi: https://doi.org/10.1101/2025.07.24.666609; this version posted July 31, 2025. The copyright holder for this preprint (which was not certified by peer review) is the author/funder, who has granted bioRxiv a license to display the preprint in perpetuity. It is made available under aCC-BY 4.0 International license.

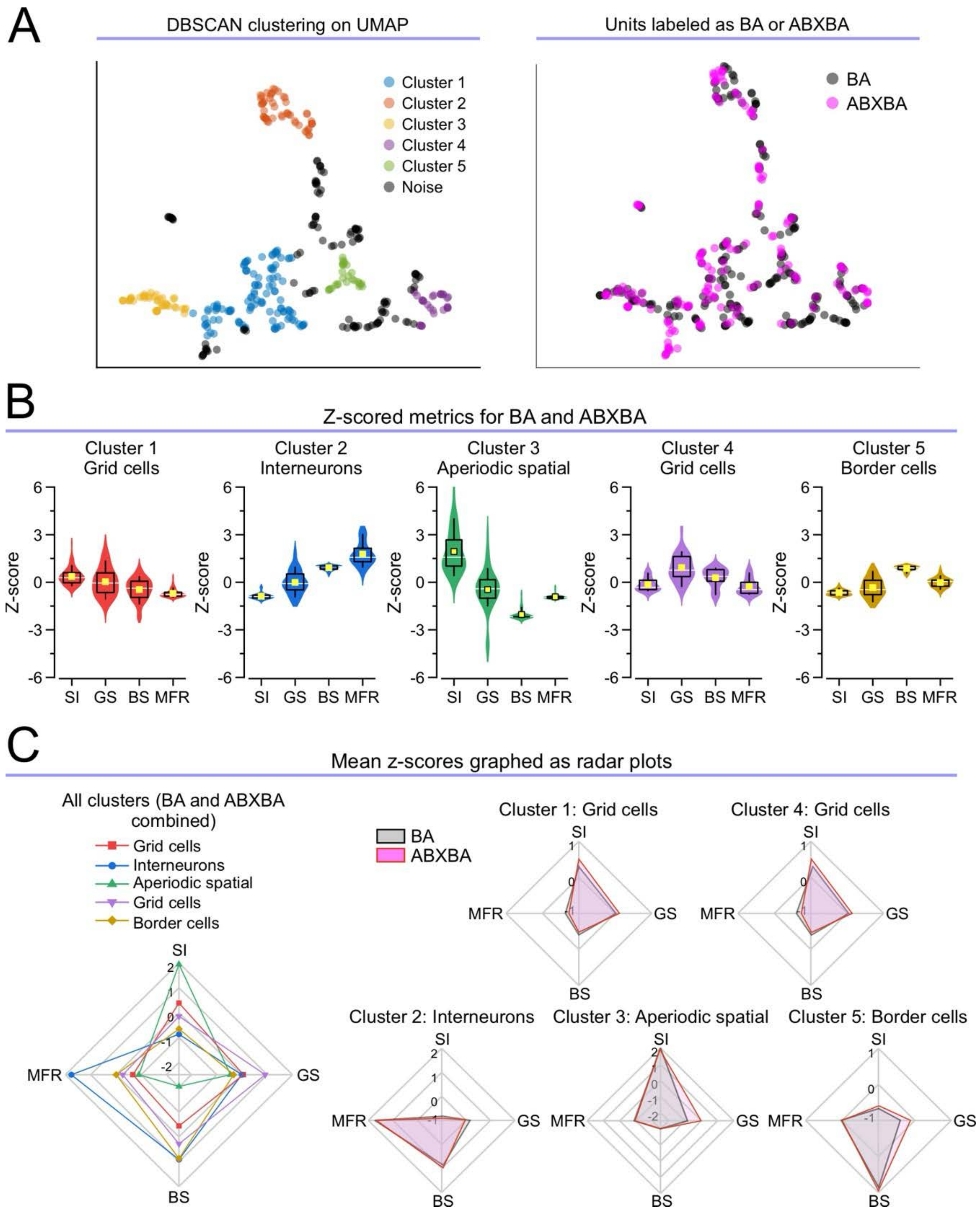

Supplemental Figure 6. Medial entorhinal cortex neurophysiology analysis pipeline in butyrate-treated mice. (A) *Left,* UMAP with DBSCAN clustering. *Right,* UMAP showing group (BA or ABXBA) for each unit. **(B)** Violin plots showing z-scored metrics for cluster classification. The clusters are identified as: grid cells (cluster 1, high grid score, most populous cluster), interneurons (cluster 2, high MFR, low SI), aperiodic spatial (cluster 4, high SI), grid cells (cluster 4, high grid score), and border cells (cluster 5, high border score). **(C)** Radar plots showing the mean SI, grid score, border score, and MFR for each cluster. In the leftmost plot, the means are shown for each cluster with BA and ABXBA mice combined. The remaining plots each show one cluster comparing BA and ABXBA mice.
